# Caffeine Consumption Patterns, Health Impacts, and Media Influence: A Narrative Review

**DOI:** 10.7759/cureus.86215

**Published:** 2025-06-17

**Authors:** Mohammed Qasim Rauf, Liyah Sharma, Edidiong Essiet, Osman Elhassan, Renad Fahim, Francis Irem-Oko, Jayadevan Sreedharan

**Affiliations:** 1 Trauma and Orthopaedics, The Hillingdon Hospitals NHS Foundation Trust, London, GBR; 2 Emergency Medicine, University Hospitals of Leicester NHS Trust, Leicester, GBR; 3 Trauma and Orthopaedics, Royal National Orthopaedic Hospital, London , GBR; 4 Medicine, Jinnah Sindh Medical University, Karachi, PAK; 5 Trauma and Orthopaedics, Leeds Teaching Hospital NHS Trust, Leeds, GBR; 6 Epidemiology and Biostatistics, Gulf Medical University, Ajman, ARE

**Keywords:** caffeine consumption, cognitive performance, global patterns, health benefits, health risks, media influence, prevalence

## Abstract

Caffeine is widely recognized as the most commonly consumed psychoactive substance globally. This review critically evaluates the physiological, psychological, and societal aspects of caffeine consumption, including the influence of media and evolving global consumption patterns. Key areas of focus include caffeine’s potential cognitive and physical effects, such as influences on memory, mood, and physical performance, alongside commonly discussed concerns such as dependence, sleep disturbances, and cardiovascular implications. The review explores how high levels of caffeine intake may lead to adverse health outcomes and considers the influence of media and advertising in shaping consumption behaviors, especially amongst younger populations and in rapidly developing regions. The review highlights the role of media in normalizing caffeine use and its potential impact on consumer behavior. The implications of these trends suggest a pressing need for further research into the long-term health consequences of caffeine consumption and for public health strategies that address the risks of overconsumption, particularly in high-risk demographics.

## Introduction and background

Caffeine is a widely consumed natural stimulant that plays a significant role in daily life across the globe. Belonging to the methylxanthine class, caffeine functions as a competitive antagonist of adenosine receptors, thereby reducing fatigue and enhancing alertness [1]. It is commonly found in plant-based sources such as coffee beans, tea leaves, cocoa, and kola nuts [2]. In addition to natural sources, caffeine is also synthetically incorporated into products like soft drinks and energy beverages to enhance energy and cognitive performance [3,4].

The cultural significance of caffeine dates back centuries, with folklore attributing its discovery to a goat herder in Ethiopia and a Chinese emperor who encountered tea serendipitously. Today, caffeine plays a vital role in modern dietary habits and cognitive functioning [1].

Contemporary consumption patterns show coffee as the most frequently consumed caffeinated beverage, driven by motivations such as sleep deprivation, academic demands, stress, and social habits [5]. Caffeine's appeal lies in its broad range of effects. Cognitively, it enhances memory, attention, concentration, decision-making, and verbal fluency. Physically, it improves endurance, strength, reaction time, and thermogenesis, while also elevating mood and promoting relaxation [6]. Research by Einöther and Giesbrecht found that caffeine boosts fat metabolism by stimulating glycogenolysis, improves attention, and task performance [7]. Furthermore, caffeine intake has been linked to the alleviation of chronic headaches [8].

However, excessive consumption can lead to adverse effects. These include symptoms associated with caffeinism, such as anxiety, insomnia, irritability, and palpitations, and may correlate with other substance use, such as tobacco [9,10]. Although acute intake improves alertness, prolonged use may result in dependency and diminished effectiveness over time [11].

This review aims to provide a comprehensive analysis of caffeine consumption, focusing on its physiological and psychological effects, health benefits, and associated risks. In addition, it explores global consumption patterns, with particular attention to the influence of media and cultural trends on caffeine use. The review is structured as follows: it first examines caffeine’s sources, followed by a discussion of media influence and global trends. Subsequently, it explores health-related outcomes, factors influencing its consumption, and directions for future research.

## Review

Sources of caffeine

Caffeine is a nitrogen-containing alkaloid that has been found in over 60 plant species. The most common sources include coffee beans, kola nuts, cocoa beans, and tea leaves [11]. They are incorporated into several kinds of products such as americano, espresso, latte, cappuccino, tea, iced tea, soft drinks, energy drinks, chocolate, chocolate milk, cocoa powder, guarana, and caffeine supplements [12]. However, evidence indicates that chocolate typically contains less caffeine compared to hot drinks such as coffee or tea [13,14]. Their caffeine content varies significantly depending on the species of coffee bean. For example, *Coffea canephora *(Robusta) has a higher caffeine concentration than *Coffea arabica *(Arabica). Additionally, the serving size, method of preparation, and quantity of coffee beans used also affect a beverage’s total caffeine content [2,11].

Prevalence of caffeine consumption

Geographical Patterns

The prevalence of caffeine consumption varies significantly by region and demographic factors, including age group and beverage preference [15]. For instance, adolescents in the USA tend to favor coffee [16], whereas tea is more commonly consumed in Asia [17]. However, Japan has seen a notable increase in coffee consumption [18]. A 2014 study reported that 85% of individuals consumed coffee daily [19]. More recently, a 2022 study by the International Food Information Council found that over 90% of American adults drink caffeinated beverages, while a 2023 survey by the Sleep Foundation indicated that 94% of Americans consume caffeine, suggesting an upward trend since 2014 [20,21]. In China, a study conducted in 2023 observed that only 5% of adults consumed caffeinated beverages from 2004 to 2018, but this percentage rose 27-fold over the 14-year period [22]. Similarly, a 2024 study in Korea found that adolescent caffeine consumption increased from 3.3% in 2015 to 12.2% in 2019 [23]. In North Lebanon, research revealed that 97% of participants drank caffeinated beverages, with specific preferences as follows: Nescafé^®^ (19.7%), chocolate (19.2%), soda (15.3%), tea (13.6%), coffee (9.8%), energy drinks (6.7%), and espresso (3.3%) [24]. In Saudi Arabia, a 2023 study focused on adolescents found that nearly 94% of participants consumed caffeinated beverages, with Arabic coffee being the most preferred [25].

Demographic Differences

In Ohio, a study found no significant difference in caffeine consumption between male and female participants [26]. However, a 2019 study revealed that a higher percentage of females consumed caffeinated beverages compared to males, 85% vs. 71% [27]. A study conducted in the USA reported that caffeine consumption increases with age, particularly among people aged 50-64, who consume an average of 165 mg per day, with coffee being the primary source of caffeine intake among older individuals. While the overall number of caffeine users has risen, the types of caffeinated beverages consumed have been shown to have evolved over time [28]. A comparative study showed a decline in coffee consumption, stable tea consumption, and an increase in energy drinks and carbonated soft drinks, especially among younger age groups [29]. Recently, caffeine consumption has become more common among older age groups in the Mediterranean region, according to a study by Torres-Collado et al. [30]. In a study conducted in Massachusetts, age was found to be a significant factor influencing caffeine intake. As students advance through their academic years, their caffeine consumption tends to increase, likely in response to more demanding study schedules. Senior-level students consumed an average of 1,698.02 mg of caffeine per week, compared to freshmen, who consumed around 1,106.23 mg weekly, equivalent to roughly six to seven cups of brewed coffee per day [29]. In a study conducted in North Lebanon, 40% of participants aged 24 and older were classified as heavy consumers, drinking more than three cups of coffee per day [24].

Daily and Weekly Consumption Trends

In a study conducted in the United States, caffeine intake was the greatest between 06:00 and 09:00 and declined progressively throughout the day. 70% of daily intake of caffeine was consumed between 03:00 and the afternoon, and about 40% of the daily intake was ingested between 06:00 and 09:00. The intake of caffeine was relatively low, totaling 7% to 8%, between 18:00 and 21:00 [31]. A separate study conducted by Martyn et al. tracked caffeine consumption throughout the week, dividing it into morning, afternoon, and evening use. Among participants aged 13 and older, the findings showed that 61% of caffeine was consumed in the morning, 21% in the afternoon, and 18% in the evening. Contrary to this, adolescents ages 13 to 17 years had a more even distribution of caffeine intake during the day, totaling 33.33% in the morning, 33.33% in the afternoon, and 33.33% in the evening [32].

Media influence on caffeine consumption

In current society, having a “24/7 lifestyle” has become the norm. To support this demanding routine, energy drinks have been heavily advertised by the media to adolescents, suggesting that they can help them stay awake, whether for studying or socializing [33,34]. However, excessive consumption poses health risks such as headaches, restlessness, heart injury, and poor sleep quality, as reported in a study conducted by Evans et al. [35].

Furthermore, spending time in coffee shops has become a prevalent trend, with marketing generating an appealing environment for social interaction, which has resulted in an increase in the purchase of caffeinated drinks [36]. A report by *Medical News Today *shows caffeine can provide protective benefits against Type 2 Diabetes, liver disease, Parkinson’s disease, liver cancer, and cardiovascular disease [37]. Media sources, including newspapers, online articles, and advertising, often emphasize caffeine’s purported benefits, which may lead the public to downplay its risks [38].

Marketing strategies play a pivotal role in shaping caffeine consumption behaviors, particularly among adolescents and young adults. A study conducted in Canada found that 83% of individuals ages 12 to 24 had been exposed to energy drink advertisements through various channels, including television, digital media, and retail environments. Exposure to sports-themed advertisements was especially influential; 71% of respondents who viewed such content believed it promoted the consumption of energy drinks during athletic activities, suggesting that marketing may normalize or encourage caffeine use in specific social contexts. Additionally, individuals with higher exposure to these advertisements were more likely to interpret them as being directed at their age group and as explicitly encouraging caffeine intake [39].

Recent evidence suggests that digital marketing via social media platforms exerts a stronger influence on energy drink consumption among young adults than traditional media. Findings from a cross-sectional survey indicate that exposure to online promotional content significantly impacts young adults’ attitudes, perceived social norms, and behavioral control regarding energy drink consumption, a core construct of the Theory of Planned Behavior [40].

An investigation into energy drink marketing on Canadian social media platforms revealed that companies employed viral marketing strategies and youth-oriented content across social media channels. In 2020, two brands were responsible for nearly 74% of all energy drink-related posts on Twitter, collectively reaching over 60% of users on the platform. Similarly, one brand accounted for more than 81% of the total user reach on Instagram and Facebook, demonstrating a dominant presence in the digital marketing space. These findings suggest that such targeted and pervasive marketing strategies may substantially increase youth engagement and contribute to the normalization and reinforcement of caffeine consumption among adolescents [41].

A study conducted in Saudi Arabia reported that approximately 82% of adolescents consumed energy drinks one to two times per week, with 42% identifying social media as their primary source of information regarding these products. The study found a significant positive correlation between social media use and energy drink consumption (r=0.592), suggesting that digital content plays a considerable role in shaping adolescents’ consumption behaviors. Moreover, the average score on the Social Media Effects Scale was 5.75 out of 8, further highlighting the considerable influence of social media exposure on adolescents’ attitudes and behaviors related to energy drink use [34].

In Canada, over 80% of youth and young adults reported seeing energy drinks marketing through various ways such as retail signage, television, and online advertisements, while 32% recalled encountering educational messages about the associated negative health effects. The most frequent educational content was schools, 16.2%, and online platforms, 15.0%. The disparity highlights the disproportionate reach of commercial marketing relative to public health information, raising concerns about the effectiveness of health education [38].

Even short-term engagement with digital marketing can have a significant influence on young adults’ intentions to consume energy drinks. Despite cognitive maturity, respondents were responsive to central persuasive cues, for instance, corporate social responsibility campaigns, which were interpreted as more effective than emotional appeals. The evidence presented suggests that digital food and beverage marketing warrants stricter regulation to reduce its impact on caffeine consumption behaviors [42].

Health benefits of caffeine consumption

Caffeine relates to countless benefits such as enhanced physical performance, ameliorated mental performance, and improved memory [1]. Research conducted by Sherman et al. exhibited that caffeine intake impacts memory in a positive manner [43]. Furthermore, research carried out at Johns Hopkins University in the United States indicated that caffeine in the form of tablets assists in the consolidation of memory [44]. Research in 2020 stipulated that caffeine intake can enhance mood. Moreover, when consumed with carbohydrates, caffeine intake significantly replenishes levels of glycogen in muscle after physical activity [45]. Caffeine can also support liver detoxification [46]. Regarding physical performance, consumption of caffeine was found to reduce muscle soreness and inflammation after physical activity [47]. Moreover, caffeine has been connected to a reduced possibility of neurodegenerative diseases, including Alzheimer’s and Parkinson’s disease [48]. A study conducted, examined the association between the consumption of caffeine and the risk of developing skin tumors in UV-treated mice. This showed that caffeine consumption resulted in inhibitory effects on carcinogenesis [49]. Caffeine has also been shown to provide relief for asthmatic patients by dilating airways, thus facilitating easier breathing [50]. Consuming caffeine capsules has also shown notable improvements in cognitive ability and mood compared to placebo groups [51].

Health risks of caffeine consumption

Adverse Effects of Caffeine Consumption

Caffeine is one of the most widely consumed legal psychoactive substances in the world and remains largely unregulated. Caffeine stimulates the central nervous system, with psychoactive properties that modify behavior like cocaine and amphetamines [52]. Caffeine is an addictive substance; once the body becomes dependent on it, it may become difficult to cease consumption. Due to this aspect, consumers may find it difficult to put an end to caffeine intake altogether. It is advisable to slowly reduce consumption by weaning doses, which can lessen the potential withdrawal symptoms such as irritability, anxiety, headache, and dizziness. Caffeine consumption is also known to result in a rise in blood pressure, which may put hypertensive or cardiac patients at risk. In some cases, caffeine overdose may lead to complications such as arrhythmias or convulsions, which could potentially be fatal [53].

High caffeine intake can disrupt the body's ability to metabolize calcium, which could then result in its deficiency, thus increasing the risk of diseases such as osteoporosis. Whilst caffeine improves alertness and energy, these effects are temporary. With time, a tolerance and dependence on caffeine can develop, resulting in fatigue and lethargy upon its cessation. Withdrawal symptoms can include fatigue, difficulty concentrating, headaches, as well as anxiety or depression. It can also exacerbate acid reflux due to its nature of stimulating gastric acid, which can aggravate heartburn. Furthermore, caffeine’s diuretic properties may worsen urinary incontinence [54]. Certain types of caffeine have been associated with a rise in cholesterol [55].

Impact of Caffeine on the Perinatal Period

A prospective cohort study conducted by Gleason et al. reported that low to moderate caffeine intake during early pregnancy correlated with indicators of reduced foetal growth, including lower birth weight, shorter crown-heel length, and decreased head and thigh circumferences. These associations were observed at intake levels below the current recommended threshold of 200 mg per day. The authors propose that caffeine may impair fetal development through physiological mechanisms such as uteroplacental vasoconstriction, which can restrict nutrient and oxygen delivery. These findings underscore the need to re-evaluate existing guidelines regarding caffeine intake during pregnancy [56].

A recent study by Hirani and Souter has highlighted potential risks associated with high caffeine intake during breastfeeding. It showed that daily caffeine consumption below 300 mg is generally safe for most lactating mothers. However, intake exceeding 450 mg per day has been linked to adverse outcomes such as infant irritability, sleep disturbances, and decreased iron concentration in breast milk, potentially increasing the risk of anemia in exclusively breastfed infants. Healthcare providers should be consulted to provide individualized guidance based on maternal consumption patterns and infant response [57].

Impact of Caffeine on Sleep

Consuming caffeinated drinks prior to sleep can negatively influence sleep patterns and quality. Caffeine decreases rapid eye movement (REM) sleep, inhibiting emotional recovery and overall rest, which is necessary for optimal functioning the next day [58]. Gardiner et al. also elucidate that higher plasma caffeine levels prior to bedtime can dysregulate sleep, leading to reduced sleep duration and potential sleep deprivation [59]. In Pfaff’s study, a significant factor considered was the relationship between caffeine consumption levels (classified as non, low, moderate, high, and very high consumers) and sleep duration. Most participants in the high and very high caffeine consumption categories reported sleeping less than seven hours per night [26]. Sleep deficiency has also been shown to be associated with increased stress and adverse effects, including mood changes, reduced performance, alertness, attention, muscle weakness, tremors, and coordination issues [60].

Long-term Health Implications

Research conducted by Cole et al. indicated that caffeine intake could elevate cortisol levels, which, over time, may impair immune system responses [61]. Although caffeine is often used to prevent fatigue and offset sleep deprivation, it may have adverse health effects. Mednick et al. suggest that caffeine increases hippocampal acetylcholine levels, potentially impairing memory consolidation by disrupting memory replay [62].

Excessive caffeine consumption is linked to higher levels of anxiety and stress, while lower doses generally have no significant impact on anxiety. Withdrawal or sleep deprivation can worsen mood and psychomotor performance. As a result, individuals may experience flu-like symptoms such as nausea and headaches, often exacerbated by high caffeine intake, insufficient sleep, or an unhealthy lifestyle [63].

Withdrawal Symptoms

Caffeine acts on two adenosine receptors (A1 and A2a), which are associated with anxiogenic effects, as shown in previous studies [64]. It also activates the hypothalamic pituitary adrenal (HPA) axis, making caffeine cessation challenging [65]. Regarding withdrawal, a study revealed that 35.4% of participants reported no symptoms, while 64.6% experienced issues such as nervousness or aggression (17%), hot flushes (13%), insomnia (12.6%), gastrointestinal issues (10.3%), shortness of breath (6.3%), and tachycardia or arrhythmia (5.4%). Over half (55.1%) could abstain from caffeine for only 2-3 days [24]. Sajadi-Ernazarova and Hamilton emphasized that caffeine withdrawal often leads to fatigue, headaches, mood declines, and irritability. To mitigate these effects, those managing caffeine dependence should reduce intake gradually [66,67].

Studies exploring the effects of caffeine intake on migraines have produced inconsistent results. Although caffeine can remedy acute migraine attacks, uncontrolled consumption has been connected to the development and frequency of migraines [68]. Caffeine consumption and withdrawal are linked to deprivation symptoms, including irritability, blurred vision, depression, fatigue, flu-like symptoms, and cognitive impairment [69].

Research on caffeine's impact on migraines has shown mixed results. While caffeine can help relieve acute migraine attacks, excessive intake is associated with increased migraine frequency. A study by Lee et al. found that caffeine abstention enhanced the effectiveness of migraine treatments and reduced headaches as assessed by the Headache Impact Test (HIT-6) [70]. Additionally, a study by Alstadhaug et al. noted that caffeine withdrawal could increase sensitivity to adenosine in migraine sufferers, reducing the threshold for migraine attacks [71].

Factors influencing caffeine consumption

Various factors contribute to caffeine consumption. The following sections elaborate on the motivations and influences related to caffeine use, supported by recent research findings.

Motivations for Caffeine Consumption

A study conducted in North Lebanon identified several reasons for the consumption of caffeinated beverages. The three primary motivations were to improve alertness (22.3%), to work long shifts alongside studying (17.6%), and for taste preference (16.7%). Additional reasons included enhancing mood, social interaction, treating migraines, and promoting relaxation. Notably, 63% of students reported an increase in caffeine consumption during stressful periods, particularly around exams, with 49.3% favoring morning consumption [24].

Sleep-Related Motivations for Caffeine Use

A significant motivation for energy drink consumption among college students is to prevent sleepiness and stay alert, reflecting a sleep-related drive to combat daytime fatigue. Mastin et al. found that 42% of participants reported using energy drinks specifically to stay alert, while 41% consumed them to prevent sleepiness. These motivations highlight how caffeine, a primary ingredient in energy drinks, is often consumed as a tool to counteract feelings of drowsiness and improve productivity during waking hours. However, while energy drinks are frequently consumed for their alerting effects, the study also revealed that frequent consumption was associated with poorer sleep quality, shorter sleep duration, and increased daytime sleepiness. This suggests that, despite the initial motivation to combat sleepiness, excessive energy drink consumption may paradoxically contribute to sleep-related issues over time [72].

Cognitive and Psychological Effects of Caffeine

Caffeine is known to influence mood, with effects such as feelings of elation and relaxation. A study by Rodak et al. showed that caffeine has concentration-dependent, non-enzymatic antioxidant properties, reducing free radical production and oxidative stress [54]. Among students, caffeine consumption significantly increases during exam periods, with 67% reporting higher intake. Norton et al.'s study found that 26% of students regularly used caffeine to prepare for exams and 27% for school projects [29]. Research by Loke highlighted a positive correlation between caffeine consumption and academic performance, with about one-third of students believing caffeine improved study efficiency [73]. Similarly, another study noted that 67% of students increased caffeine intake during exams to boost cognitive function, linked to caffeine's stimulatory effects on the central nervous system [51]. A similar trend was observed in Lebanon, where caffeine consumption rose in tandem with academic stress. Despite understanding caffeine's potential downsides, 73% of participants did not perceive it as harmful [24]. Jamil et al. also expressed that 67% of students increased caffeine intake during examinations to intensify cognitive function. This association derives from caffeine’s stimulatory effects on the central nervous system, causing an amplification of neurotransmission [74].

## Conclusions

This article has reviewed several aspects of caffeine consumption, such as its sources, prevalence, health benefits, health risks, and the influence of media on its consumption. Caffeine is derived from various plant sources and is increasingly prevalent in modern diets, playing a staple role in daily life, particularly among adults and students. The impact of media and marketing on caffeine consumption trends cannot be understated, as advertisements have shaped perceptions of caffeine as a quick fix for fatigue and a means of enhancing productivity.

The benefits of caffeine, such as improved physical performance, cognitive function, and preventative effects against several diseases, provide a convincing case for its consumption. However, these benefits come with risks such as addiction, cardiovascular issues, and detrimental effects on sleep. Demographic data reveal differing consumption patterns among age groups and regions, with increases among adolescents and a preference for energy drinks and coffee. Despite extensive literature on caffeine’s immediate effects, longitudinal studies on cumulative health outcomes, especially in younger populations, remain limited. Future research should explore culturally specific consumption patterns, long-term sleep implications, and the role of emerging media platforms in shaping health behaviors.
